# Structure Formation and Regulation of Au Nanoparticles in LiTaO_3_ by Ion Beam and Thermal Annealing Techniques

**DOI:** 10.3390/nano12224028

**Published:** 2022-11-16

**Authors:** Yong Liu, Xinqing Han, Jinhua Zhao, Jian Sun, Qing Huang, Xuelin Wang, Peng Liu

**Affiliations:** 1 Institute of Frontier and Interdisciplinary Science and Key Laboratory of Particle Physics and Particle Irradiation (MOE), Shandong University, Qingdao 266237, China; 2 School of Science, Shandong Jianzhu University, Jinan 250101, China; 3 Shanghai Institute of Applied Physics, Chinese Academy of Sciences (CAS), Shanghai 201800, China

**Keywords:** nanoparticles, lithium tantalate, ion implantation, swift heavy ion irradiation, size and spatial distribution

## Abstract

The size uniformity and spatial dispersion of nanoparticles (NPs) formed by ion implantation must be further improved due to the characteristics of the ion implantation method. Therefore, specific swift heavy ion irradiation and thermal annealing are combined in this work to regulate the size and spatial distributions of embedded Au NPs formed within LiTaO_3_ crystals. Experimental results show that small NPs migrate to deeper depths induced by 656 MeV Xe^35+^ ion irradiation. During thermal annealing, the growth of large Au NPs is limited due to the reductions in the number of small Au NPs, and the migrated Au NPs aggregate at deeper depths, resulting in a more uniform size distribution and an increased spatial distribution of Au NPs. The present work presents a novel method to modify the size and spatial distributions of embedded NPs.

## 1. Introduction

Due to their surface, small size, quantum size, and quantum tunneling effects, metallic nanoparticles (NPs) are widely used in basic research fields, including photonics, biology, chemistry, energy, and information science [1,2,3,4,5]; they play a significant role in practical application fields as catalysts, and as components of optical sensors, waveguide devices, molecular detectors, and biomedical technologies [6,7,8,9,10]. Ion implantation technology is an effective method for fabricating embedded NPs, which can break the solid solubility limits of various materials [11]. The synergistic effect between NPs fabricated by the ion implantation method and the host material shows many excellent properties, such as localized surface plasmon resonance [12], high-order nonlinear enhancement and modulation [13].

Previous research has focused on embedding NPs in amorphous silica [14,15], while crystalline materials, such as LiNbO_3_ and LiTaO_3_, are proven to be emerging platforms for forming NPs [16,17]. By considering the ubiquity and versatility of functional crystalline materials, the purposeful design of nanostructures in crystals (such as LiTaO_3_) through the use of ion beams and comprehensive exploration of the physical mechanisms can facilitate the development of related applications. In addition, the NP performance is closely associated with shape, size, and spatial distribution. Due to the characteristics of the ion implantation method, the size uniformity and spatial dispersion of NPs are not controlled, and further improvement is needed. During the ion implantation process, by adjusting the implantation conditions, the spatial distribution of NPs can be controlled to a certain extent [18,19]. By separating the nucleation and growth processes of NPs, shortening of the nucleation process has been shown to induce a narrow spatial distribution of the formed NPs [20,21]. Over the past several decades, energetic ion irradiation has been considered a useful tool for material analysis, material modification, and nanoscale processing [22,23,24,25,26]. Defects induced by low-energy ion irradiation are used as prenucleation centers of NPs, resulting in the regulation of the NP nucleation and distribution [27,28]. Meanwhile, swift heavy ion (E/M > 1 MeV/u) irradiation with high electronic energy loss is proven to be an efficient ion beam technology to reshape the morphologies of NPs [29]. Under swift heavy ions, once the electronic energy loss exceeds a certain threshold, energetic ions create ion tracks along their trajectories [30,31]. An NP shape elongation behavior is induced by swift heavy ion irradiation only for those NPs larger than the ion track diameter [32].

In this work, by irradiating LiTaO_3_ crystals with embedded Au NPs with 656 MeV Xe^35+^, in combination with thermal annealing, the size and spatial distributions of Au NPs are effectively regulated, and the mechanisms of tailoring the size and spatial distribution of NPs are further discussed.

## 2. Materials and Methods

Single-crystal LiTaO_3_ samples with a (006) surface normal zone axis directions and 10 × 10 × 1 mm^3^ dimensions were used in this work. Three sets of ion implantation or irradiation experiments were conducted. (i) The LiTaO_3_ sample was implanted with 200 keV Au^+^ ions with a fluence of 3 × 10^16^ cm^−2^ at 300 K. (ii) The LiTaO_3_ sample was irradiated with 656 MeV Xe^35+^ ions with a fluence of 3 × 10^11^ cm^−2^ at 300 K. (iii) The LiTaO_3_ sample was first implanted with 200 keV Au^+^ ions at 300 K and subsequently irradiated with 656 MeV Xe^35+^ ions at 300 K. In this work, Au^+^ ion implantation was performed using an implanter at the Institute of Semiconductors, Chinese Academy of Science. Swift Xe^35+^ ion irradiation was conducted at the Heavy Ion Research Facility in Lanzhou (HIRFL), Institute of Modern Physics, Chinese Academy of Sciences. In addition, the Au^+^-implanted sample and the Au^+^-implanted and subsequently Xe^35+^-irradiated sample were annealed in a furnace at 1073 K for 60 min in an Ar gas environment.

TEM, high-resolution TEM (HRTEM), energy dispersive X-ray spectroscopy (EDS) line scan, and high-angle annular dark-field scanning TEM (HAADF–STEM) were used to characterize the microstructures of LiTaO_3_ specimens with different irradiation conditions. Cross-sectional TEM samples were prepared using an ion milling process performed on a focused ion beam mill in addition to a lift-out process performed on an FEI Helios NanoLab 600 Dual Beam system (Hillsboro, OR, USA). The TEM images were obtained with an FEI Tecnai G2 F20 transmission electron microscope (Hillsboro, OR, USA). Notably, the TEM observation time for each sample was relatively short and would not induce recrystallization of the lattice damage formed under electron beam irradiation [33].

The electronic energy loss of LiTaO_3_ crystal and bulk Au induced by 656 MeV Xe^35+^ irradiation were determined through the Stopping and Range of Ions in Matter (SRIM) 2013 full-cascade simulation code [34], using densities of 7.45 g cm^−3^ for LiTaO_3_ and 19.31 g cm^−3^ for Au and threshold displacement energies of 25 eV for Li, 25 eV for Ta, 28 eV for O sublattice. The iTS model was used to numerically calculate the spatiotemporal evolution of the energy deposition and the lattice temperature induced by the electronic energy loss under swift Xe^35+^ ion irradiation [35,36]. The iTS model describes the energy transfer from the incident ion to the target material. In this model, the system is treated as two coupled subsystems: the electron and lattice subsystems. The energy exchange between the two subsystems is described by a set of coupled thermal diffusion equations for the electron subsystem (Equation (1)) and the lattice subsystem (Equation (2)).

(1) $$C_{e}\left(T_{e}\right)\frac{\partial T_{e}}{\partial t}=\frac{1}{r}\frac{\partial }{\partial r}\left[rK_{e}\left(T_{e}\right)\frac{\partial T_{e}}{\partial r}\right]-g\left(T_{e}-T_{a}\right)+A\left(r,t\right)$$

(2) $$C_{a}\left(T_{a}\right)\frac{\partial T_{a}}{\partial t}=\frac{1}{r}\frac{\partial }{\partial r}\left[rK_{a}\left(T_{a}\right)\frac{\partial T_{a}}{\partial r}\right]+g\left(T_{e}-T_{a}\right)$$

where $C_{e}$ and $C_{a}$ are the specific heat coefficients of the electron and lattice subsystems, respectively; $K_{e}$ and $K_{a}$ are the corresponding thermal conductivities; g is the electron–phonon coupling strength parameter; A(r,t) is the energy deposition from the incident ions to the electrons [37]; and $g\left(T_{e}-T_{a}\right)$ is used to model the energy exchange between the two subsystems. In addition, hot electrons in the conduction band of an insulator are expected to behave similarly hot electrons in a metal [38]. When $T_{e}$ < $T_{a}$, electrons are supposed to be confined in the lattice, preventing lattice cooling by cold free electrons [39,40]. Due to the possibility of a phase change in the material, the two differential equations can be solved only numerically [41]. In this work, $K_{a}$ and $C_{a}$ were extracted from reference [42]. $C_{e}$ was set to 1.0 J cm^−3^ K^−1^, and $K_{e}$ was set to 100 W m^−1^ K^−1^ because $K_{e}=C_{e}\times D_{e}$, where $D_{e}$ is the electron diffusivity (1.0 cm^2^ s^−1^), and g was set to 5.7 × 10^18^ W m^−3^ K^−1^ [42].

## 3. Results and Discussion

**Figure 1:**
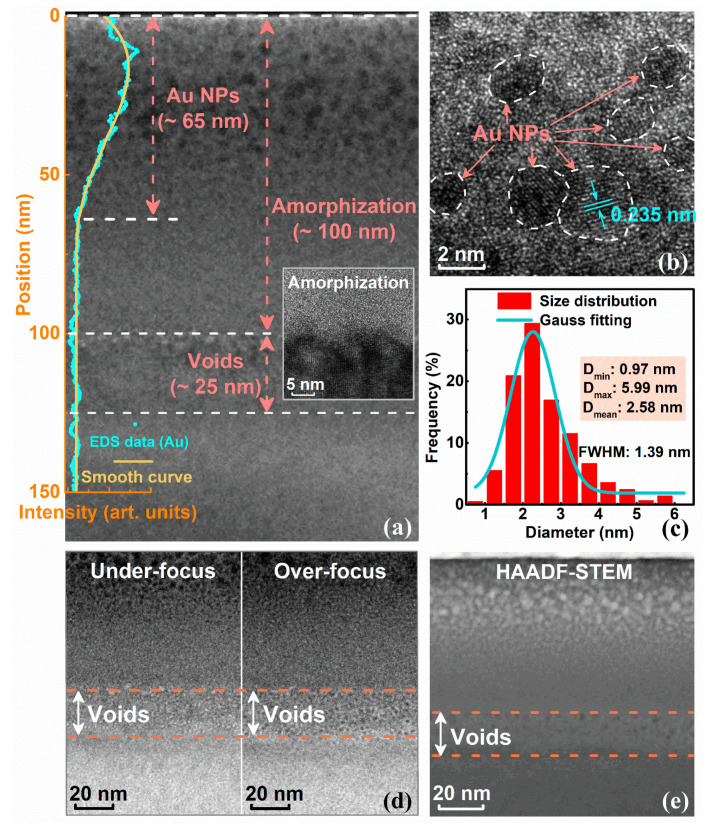
(**a**) Cross-sectional TEM image and (**b**) HRTEM image of Au NPs embedded in the LiTaO_3_ sample; (**c**) diameter distribution of synthesized NPs; (**d**) TEM images of a void region under underfocusing and overfocusing conditions; (**e**) HAADF–STEM image of Au NPs embedded in the LiTaO_3_ sample.

TEM observations were performed on sample cross-sections to visually characterize and confirm the microstructures of Au NPs embedded in LiTaO_3_ crystals. As shown in Figure 1a, in addition to an amorphous region with an approximately 100 nm thickness below the sample surface, Au^+^ ion implantation at a fluence of 3 × 10^16^ cm^−2^ leads to the formation of the Au NP region with an approximately 65 nm thickness below the sample surface. The Au elemental distribution obtained by EDS elemental line scan is also presented in Figure 1a, further confirming the spatial distribution of Au NPs. Moreover, the HRTEM image 100 nm below the surface (shown in the inset of Figure 1a) exhibits a clear amorphous-crystalline boundary. Furthermore, the HRTEM image of the Au NPs region indicates the formation of crystalline NPs with an almost spherical shape, as shown in Figure 1b. According to HRTEM observations, the interplanar distance (0.235 nm) closely fits the (111) plane of the embedded Au NPs [43]. As shown in Figure 1c, the diameters of the embedded Au NPs range from 0.97 nm to 5.99 nm, and the mean diameter is approximately 2.58 nm. Moreover, as shown in Figure 1a, a clear contrast appears at a depth range of 100–125 nm from the surface. To obtain the microstructure of the contrast region, Figure 1d was obtained by intentionally underfocusing and overfocusing the objective lens, and a large number of voids with thicknesses of 25 nm are clearly observed. The HAADF–STEM image, shown in Figure 1e, confirms the observations regarding the formation of void regions. The presence of voids provides the potential for spalling on the nanoscale; more generally, it exhibits a negative effect on the stability of the crystal structure.

**Figure 2:**
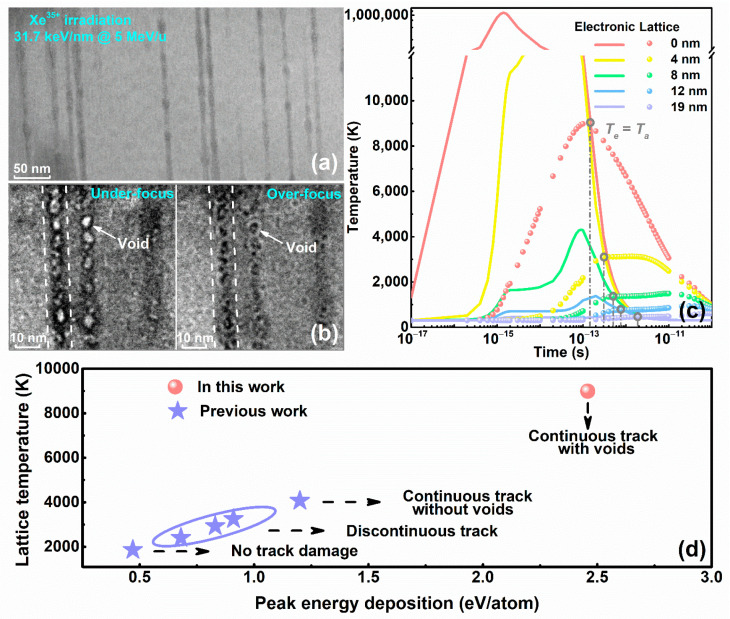
LiTaO_3_ sample irradiated by 656 MeV Xe^35+^ ions: (**a**) and (**b**) cross-sectional TEM images; (**c**) electronic temperature and lattice temperature induced by Xe^35+^ ion irradiation; and (**d**) lattice temperature of the LiTaO_3_ crystal as a function of the peak energy deposition in this work and previous work [44].

Cross-sectional TEM images taken from the surface (electronic energy loss peak region) of the 656 MeV Xe^35+^ ion-irradiated LiTaO_3_ sample are shown in Figure 2a,b. The cross-sectional TEM image in Figure 2a clearly shows the formation of continuous tracks with diameters of approximately 10 nm. In addition, by intentionally underfocusing and overfocusing the objective lens, the TEM image in Figure 2b confirms that a large number of voids exist inside the ion track. The formation mechanism for the fine structure of the track induced by 656 MeV Xe^35+^ irradiation is discussed based on the iTS model. The ion irradiation energy is immediately deposited into the electron subsystem of the sample within a time scale of ~10^−15^ s, resulting in an electron cascade via electron–electron interactions. Then, due to the difference between the electron temperature and the lattice temperature, the deposited energy is transferred from the hot electron subsystem to the cold lattice subsystem via electron–phonon interactions, resulting in a local increase in temperature. Once the electronic energy loss induced by swift Xe^35+^ ion irradiation exceeds a certain threshold, local melting occurs, and an ion track is formed along the ion trajectory in the LiTaO_3_ crystal via a subsequent rapid quenching process.

Figure 2c shows the results obtained using the iTS model for the spatiotemporal evolution of electronic temperature and lattice temperature. The electronic and lattice temperatures at the ion path center (0 nm) reach thermal equilibrium ($T_{e}=T_{a}$) at approximately 10^−13^ s, and the lattice temperature peaks at 8978 K, which is much higher than the melting temperature (1923 K). Our previous studies [44] have shown that in agreement with Figure 2d, the peak lattice temperature reaches 4070 K in the formation of amorphous continuous tracks without voids in LiTaO_3_ crystals. The peak lattice temperature in this work reaches 8978 K. The extremely high temperature of the thermal peak and the large kinetic energy of the central atom of the ion track cause sufficiently violent collisions to make all the atoms in the center of the thermal peak move far away; with the rapid quenching of the system, the atoms rapidly cool, forming discontinuous voids inside the track.

**Figure 3:**
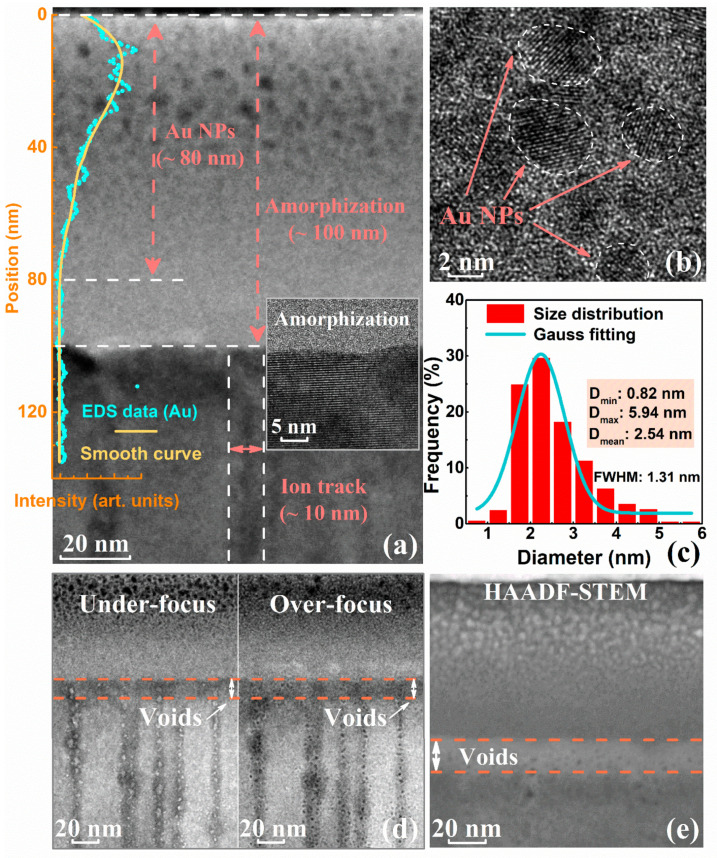
(**a**) Cross-sectional TEM image and (**b**) HRTEM image of the LiTaO_3_ sample with embedded Au NPs after subsequent Xe^35+^ ion irradiation; (**c**) diameter distribution of synthesized NPs; (**d**) TEM images of a void region under underfocusing and overfocusing conditions; and (**e**) HAADF–STEM image of the LiTaO_3_ sample with embedded Au NPs after subsequent Xe^35+^ ion irradiation.

The Au^+^-implanted LiTaO_3_ sample was then irradiated with 656 MeV Xe^35+^ ions at a fluence of 3 × 10^11^ cm^−2^. Compared to the initial Au^+^-implanted sample (Figure 1a), the TEM image (Figure 3a) clearly shows that the thickness of the amorphous region (~100 nm) does not substantially change upon subsequent Xe^35+^ ion irradiation. However, based on TEM and EDS line scan analysis, the thickness of the Au NPs region increased from 65 nm to 80 nm, indicating that Xe^35+^ irradiation induces the migration of small NPs to deeper depths. In addition, as shown in Figure 3b, the formed crystalline NPs are almost spherical in shape, with an interplanar distance of 0.235 nm. The 656 MeV Xe^35+^ ion irradiation does not induce shape elongation of the NPs due to the diameter of the track being larger than that of the NPs [32]. Notably, as shown in Figure 3c, the diameters of the embedded Au NPs range from 0.82 nm to 5.94 nm, and the mean diameter is approximately 2.54 nm; this value is slightly smaller than the NP size of the initial Au^+^-implanted sample. This is attributed to the difference in thermal peak responses of the material substrate and embedded NPs during swift heavy ion irradiation, which induces all the parts of small NP turn into melt, while most parts of the large NP do not turn into melt [45]. As shown in Figure 3d,e, the underfocused and overfocused images and HAADF–STEM image show that the void region range is smaller than that in the initial Au^+^-implanted sample, which is attributed to the partial recrystallization of defects due to the thermal spike response induced by Xe^35+^ ions irradiation.

**Figure 4:**
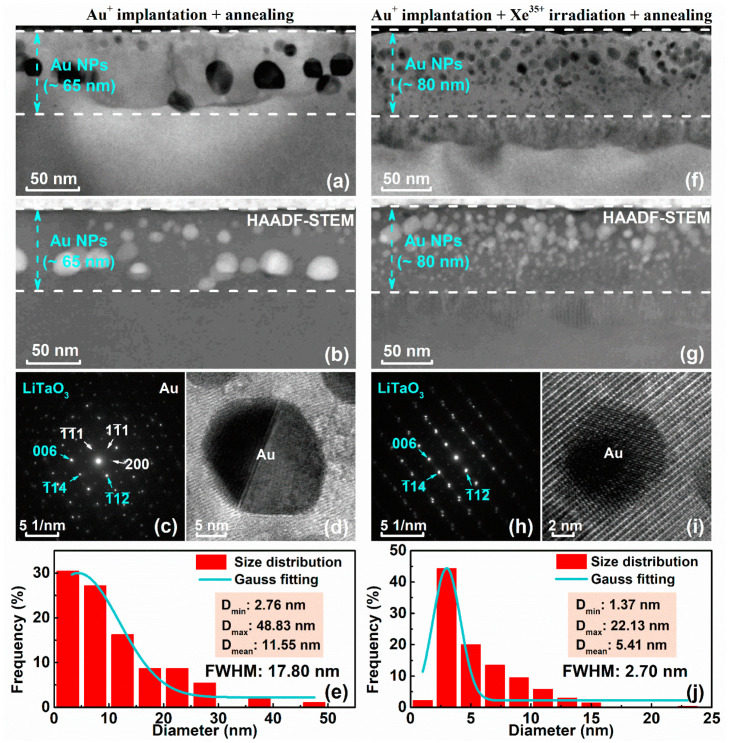
LiTaO_3_ sample with embedded Au NPs after annealing at 1073 K: (**a**) cross-sectional TEM image; (**b**) HAADF–STEM image; (**c**) SAED image; (**d**) HRTEM image; and (**e**) diameter distribution of NPs. LiTaO_3_ sample with embedded Au NPs after subsequent Xe^35+^ ion irradiation and then annealing at 1073 K: (**f**) cross-sectional TEM image; (**g**) HAADF–STEM image; (**h**) SAED image; (**i**) HRTEM image; and (**j**) diameter distribution of NPs.

Post-implantation thermal annealing after the nucleation stage accelerates aggregation of implanted atoms for further enlargement of NPs and effectively enables recovery from the damage induced by ion implantation or irradiation [46,47]. As shown in Figure 4a,b, the TEM and HAADF images of the LiTaO_3_ sample with embedded Au NPs after annealing at 1073 K show that the thickness (~65 nm) of the Au NP region is almost the same as that before annealing. However, the TEM (Figure 4f) and HAADF (Figure 4g) images of the Xe^35+^-irradiated LiTaO_3_ sample with embedded Au NPs after annealing at 1073 K show that the thickness of the NP region is significantly wider (~80 nm) than that before annealing (~65 nm). The absence of a diffraction ring in the selected area electron diffraction (SAED) images (Figure 4c,h) of the near-surface region and the ordered arrangement of the matrix lattice in the HRTEM image (Figure 4d,i) of typical NPs indicate that the amorphous region recrystallizes after annealing. Furthermore, for the embedded Au NP sample after annealing at 1073 K, as shown in Figure 4e, the smallest NP size is only 2.76 nm and the largest size reaches 48.83 nm. Correspondingly, as shown in Figure 4j, the NP size of the Xe^35+^-irradiated LiTaO_3_ sample with embedded Au NPs after annealing at 1073 K is more uniform, and the maximum size only reaches 22.13 nm. During thermal annealing, the growth of large Au NPs is limited due to the reduction in the number of small Au NPs around the large Au NPs induced by Xe^35+^ ion irradiation, eventually inducing a more uniform size distribution of Au NPs. Furthermore, Xe^35+^ ion irradiation induces the migration of small NPs to deep depths, leading to the aggregation of Au NPs at high temperatures, increasing the broadened distribution of NPs. The measurement results prove that the size and spatial distribution of Au NPs embedded in LiTaO_3_ crystals are effectively regulated by combining specific swift heavy ion irradiation and thermal annealing.

## 4. Conclusions

In this work, the size and spatial distribution of Au NPs embedded in LiTaO_3_ crystals were effectively regulated by the specific swift ion irradiation combined with thermal annealing. Embedded Au NPs were formed within the LiTaO_3_ crystal using 200 keV Au^+^ ion implantation and subsequently irradiated with 656 MeV Xe^35+^, where the diameter of the track induced by Xe^35+^ irradiation was larger than that of the Au NPs. In addition, small NPs migrated to deeper depths induced by Xe^35+^ irradiation. During thermal annealing, the growth of large Au NPs was limited due to the reduction in small Au NPs, and the migrated Au NPs aggregated in deep layers, resulting in a more uniform size distribution and an increased spatial distribution of Au NPs. This work could provide a novel tool and a powerful method for modifying the size and spatial distribution of NPs, which can be exploited in future devices for novel applications.

## Data Availability

Data will be available upon request from the corresponding authors.
